# Keep it simple? Predicting primary health care costs with clinical morbidity measures

**DOI:** 10.1016/j.jhealeco.2014.02.005

**Published:** 2014-05

**Authors:** Samuel L. Brilleman, Hugh Gravelle, Sandra Hollinghurst, Sarah Purdy, Chris Salisbury, Frank Windmeijer

**Affiliations:** a Centre for Academic Primary Care, University of Bristol, United Kingdom; b Centre for Health Economics, University of York, United Kingdom; c Department of Economics, University of Bristol, United Kingdom

**Keywords:** Primary care, Costs, Horizontal equity, Capitation, Risk rating

## Abstract

Models of the determinants of individuals’ primary care costs can be used to set capitation payments to providers and to test for horizontal equity. We compare the ability of eight measures of patient morbidity and multimorbidity to predict future primary care costs and examine capitation payments based on them. The measures were derived from four morbidity descriptive systems: 17 chronic diseases in the Quality and Outcomes Framework (QOF); 17 chronic diseases in the Charlson scheme; 114 Expanded Diagnosis Clusters (EDCs); and 68 Adjusted Clinical Groups (ACGs). These were applied to patient records of 86,100 individuals in 174 English practices. For a given disease description system, counts of diseases and sets of disease dummy variables had similar explanatory power. The EDC measures performed best followed by the QOF and ACG measures. The Charlson measures had the worst performance but still improved markedly on models containing only age, gender, deprivation and practice effects. Comparisons of predictive power for different morbidity measures were similar for linear and exponential models, but the relative predictive power of the models varied with the morbidity measure. Capitation payments for an individual patient vary considerably with the different morbidity measures included in the cost model. Even for the best fitting model large differences between expected cost and capitation for some types of patient suggest incentives for patient selection. Models with any of the morbidity measures show higher cost for more deprived patients but the positive effect of deprivation on cost was smaller in better fitting models.

## Highlights

• Clinical measures greatly increase the predictive power of primary care cost models.
• Counts of diseases perform better than complex multimorbidity measures.
• Capitation for given patient types varies strongly with the morbidity measure.
• Gains from patient selection are substantial with all morbidity measures.
• Pro poor horizontal inequity is smaller the better fitting the cost model.

## 1 Introduction

The relationship between patients’ primary care costs (consultations, tests, drugs) and their characteristics (morbidity, age, gender, socio-economic circumstances) is of interest for two reasons. First, primary care providers (general or family practitioners) are increasingly paid prospectively via capitation fees to cover the costs of patients for whom they have agreed to provide care. Examples of health care systems with capitation payments for general practice include Austria, Denmark, Ireland, Italy, the Netherlands, New Zealand, Norway, Ontario, and the UK (Boerma, 2003; European Parliament, 1998; Sibley and Glazier, 2012). Capitation payment has been advocated for primary care “medical homes” in the US (Goroll et al., 2007) and at least one US insurance scheme has experimented with capitation payments for primary care providers (Ash and Ellis, 2012; Ellis and Ash, 2012). If funders wish to ensure that patients with greater needs for health care carry a larger capitation, to reduce financial incentives for providers to cream skim or dump patients, or to give providers incentives to improve outcomes, then it is necessary to know how patients’ expected cost varies with their characteristics (Schokkaert et al., 1998; Sibley and Glazier, 2012; Ash and Ellis, 2012). Despite the prima facie importance of morbidity as a determinant of healthcare costs, most primary care capitation systems currently relate payments only to patient age and gender.

The second reason for interest in the relationship between the cost of patients and their characteristics is to investigate whether primary healthcare resources are equitably allocated. Horizontal equity requires that patients in equal need should receive equal amounts of health care. Multiple regression models of cost can be used to test whether there is horizontal equity in the allocation of primary care resources: after allowing for need, cost should not vary with a patient's socio-economic status (Gravelle et al., 2006; Bago d’Uva, 2005). But if the data on patient morbidity are poor, any association between socio-economic status and healthcare costs may not be due to horizontal inequity but to the correlation of socio-economic status and unobserved aspects of morbidity.

Electronic patient records in general practices make it possible to obtain very detailed information on the medical history of patients. The raw data are so rich^1^ that they must be aggregated to produce morbidity measures which are useful for analysis.

The simplest approach is to group diagnoses into a manageable number of morbidity categories which can then be included in regression models of patient costs as a set of dummy variables indicating the presence or absence of specific diagnoses. This assumes that the effect of diagnoses is additive. But the cost of one patient with both diabetes and depression may be greater than the cost of two patients, one with diabetes and the other with depression, because it may be more difficult to control blood sugar levels for a depressed patient. Conversely, there may be cost savings with some multimorbid patients. For example, heart disease and diabetes are conditions where monitoring of cholesterol may be required but the associated costs need only be incurred once in a given period for a patient with both conditions. Allowing for the possible non-additive effects of multimorbidity is potentially important since the proportion of the population who are multimorbid is non-trivial (20% to 61% in our data set depending on the multimorbidity measure used) and has been growing over time (Hippisley-Cox and Pringle, 2007).

The raw clinical data can be combined in many ways to produce different sets of diagnostic categories and diagnostic categories can in turn be combined in different ways to produce definitions of multimorbidity. A recent review (Huntley et al., 2012) found 17 different multimorbidity classification systems ranging from simple counts of the number of diagnoses, as in the Charlson system, to elaborate classification schemes such as the John Hopkins Adjusted Clinical Groups (ACG) Case-Mix system (Johns Hopkins Bloomberg School of Public Health, 2008) and the Hierarchical Conditions Classification (Pope et al., 2004).

The availability of different morbidity systems derived from the same raw clinical data raises a number of questions. How much do clinical measures of morbidity improve the performance of models of patient cost compared with simpler models based on age and gender? Do measures which account for multimorbidity perform better than simpler morbidity systems which do not allow for possible interactions amongst diagnoses? Does the morbidity system used affect the relative performance of different estimators? In terms of the two policy motives for estimating cost models: how do capitation payments based on detailed clinical measures differ from those based only on age and sex? Are estimates of the relationship between socioeconomic status and cost sensitive to which morbidity system is used?

In this paper we address these questions using detailed clinical data from 86,100 patients in 174 English general practices. We construct eight morbidity measures which we use in linear ordinary least squares (OLS) models and in exponential (log link, Poisson) generalised linear models (GLM). We compare the goodness of fit of these models with a basic model containing only patient age, gender, and deprivation. We calculate capitation payments based on different morbidity schemes and compare capitation payments with costs for selected types of patient. We also compare the results of a simple test of horizontal inequity using the different morbidity schemes.

Our eight morbidity measures are derived from four morbidity descriptive systems: 17 chronic diseases in the Quality and Outcomes Framework (QOF); 17 chronic diseases in the Charlson scheme; 114 Expanded Diagnosis Clusters (EDCs); and 68 Adjusted Clinical Groups (ACGs). The Charlson measures have the worst performance but still improve markedly on models containing only age, gender, deprivation and practice effects. The EDC measures perform best followed by the QOF and ACG measures. In general, for a given disease description system, counts of diseases and sets of disease dummy variables have similar explanatory power. Simple counts of EDC or QOF conditions perform better than the more elaborate ACG multimorbidity measure.

Rankings of the measures are broadly similar whether the cost model was linear (OLS) or exponential (GLM). However, the choice of morbidity measure does affect the relative performance of the two estimation methods. OLS is better than GLM with three of the morbidity measures, GLM is better with three, and for two of the measures GLM and OLS have virtually identical performance.

We use the cost models to calculate capitation payment as the cost predicted for a patient given their age, gender, deprivation and morbidity but removing the effect of the patient's practice and replacing it by the average of the practice effects. Capitation payments, at patient and at practice level, are sensitive to the choice of estimation method and morbidity measure. We also find that the difference between average cost and capitation for some types of patient is often substantial, so that there are incentives for patient selection, though less than when capitation is based only on age and gender.

Our data do not permit the construction of sophisticated measures of horizontal inequity such as the concentration index: we are limited to simple comparisons of cost for patients in different deprivation deciles. Comparison of primary care cost for the top and bottom deciles of deprivation suggests that there is pro-deprived inequity even after allowing for clinically measured morbidity. Estimates of the degree of inequity depend on the morbidity measure used in the cost model. When only age, gender, deprivation, and practice effects are included the ratio of the cost of patients in the top deprivation decile relative to those in the bottom decile is 1.50. The ratio is reduced to 1.19 when QOF indicators are added to the model and to 1.15 when EDC indicators are used. Generally, the better fitting is the morbidity model the smaller is the ratio of costs for patients in the top and bottom deprivation deciles.

### 1.1 Related literature

The ACG system has been used in studies of primary care costs and utilisation in Canada (Reid et al., 2001), Spain (Orueta et al., 1999), Sweden (Halling et al., 2006), the UK (Sullivan et al., 2005; Omar et al., 2008), and the US (Starfield et al., 1991). Ash and Ellis, 2012 applied an extended version of the Hierarchical Clinical Conditions morbidity system used in Medicare reimbursement to explain the costs that primary care patients should have incurred if managed appropriately. Some of these papers, as in Ash and Ellis, 2012 have used concurrent morbidity from the period in which costs were incurred and report *R*^2^ over 0.5. However, for a prospective capitation system it is necessary to examine how past morbidity predicts future costs. Studies using past morbidity usually find an *R*^2^ larger than 0.3, compared with an *R*^2^ below 0.1 from models using only data on patient age and gender. We build on this previous work by comparing the predictive power of the ACG system with that of the QOF and Charlson morbidity classifications.^2^

Previous studies of horizontal equity in primary care have been based on population surveys with self reported health. Bago d’Uva (2005), using the British Household Panel Survey, found that patients with higher income had more consultations after controlling for other socio-economic characteristics, and for previous period patient reported health as measured by the General Health Questionnaire, self assessed health, the number of health problems, and an indicator of whether health limited daily activities. Morris et al., 2005 used data from the Health Survey for England and found a negative but insignificant association of income and higher social class with consultations after controlling for current self reported general health, the presence of long standing illnesses, and days of acute illness. Generally as more measures of morbidity are included in the analysis the degree of pro-poor inequity falls (van Doorslaer et al., 2000). Other methods of allowing for unobserved differences in morbidity also reduce measured pro-poor inequity. Bago d’Uva et al. (2009) find that using panel data to allow for unobserved time invariant patient differences reduces the extent of pro-poor horizontal inequity in GP visits in most European countries, in some cases leading to pro-rich inequity. In Bago d’Uva et al. (2011), using vignettes to allow for reporting bias and objective indicators such as grip strength and date recall tests to instrument for self reported health reduced the association of worse education with more GP visits. Because we have a more limited measure of deprivation (ratios of cost for patients in different deprivation deciles) we do not attempt a full analysis of horizontal equity, but our study complements these previous investigations by showing that using detailed clinical data on individual patients also reduces the extent of pro-deprived inequity in use of primary care.

The payment system for primary care should take account of provider altruism, the risk imposed on providers, and the incentives for supplier inducement of demand, efficiency in production, selection of patients, and gaming of reporting. The theoretical literature suggests that a mixed payment system is likely to be optimal, combining elements of fee for service and capitation payments related to patient characteristics (see McGuire, (2011) for a summary of the arguments). We do not attempt to derive an optimal payment system but our results are relevant in that they show that the estimation of patient cost models for the capitation component will be greatly improved by including detailed clinical morbidity measures but that the choice of particular morbidity scheme will have marked effects on capitation for individual patients. We also illustrate the magnitude of potential incentives for both patient selection and gaming of reporting of patient morbidity.

The distribution of healthcare costs for individual patients usually has a long right hand tail and a spike at zero cost reflecting non-use by a non trivial proportion of the population. This has led to some debate about the appropriate estimation method for models of individual cost with suggestions including transformation of the cost variable, two part models, and Generalised Linear Models (GLMs) (Blough et al., 1999; Buntin and Zaslavsky, 2004; Manning, 2006; Manning and Mullahy, 2001; Manning et al., 2005; Mullahy, 1998). Although our main interest is in the implications of using detailed clinical morbidity information in cost models rather than econometric methods, our comparisons of GLM and OLS estimators contribute to the debate over healthcare cost estimators. Ours is the first comparison using primary care data, rather than hospital or total health care costs, and it shows that the relative performance of different estimators depends on the morbidity measure.

Section 2 describes the data and the estimation methods. The model results are set out in Section 3 and their implications for capitation discussed in Section 4. Section 5 concludes.

## 2 Methods

### 2.1 Data

#### 2.1.1 Institutional setting

To receive primary medical care in the British National Health Service (NHS) patients must register with a general (family) practice, which also acts as gatekeeper for elective hospital care. The NHS is financed almost entirely from general taxation and patients face no charges for NHS health care, apart from a small charge for drugs prescribed in primary care. Because of the wide range of exemptions on grounds of age, income, and health, around 90% of drug prescriptions carried no charge in 2007/8.

#### 2.1.2 Sample

The General Practice Research Database (GPRD) contains primary care medical records for around 5 million patients currently registered with general practices in the United Kingdom. The GPRD is broadly representative of the general population in the UK (Lawrenson et al., 1999).

An initial random sample of patients aged 18 years and over was drawn from the 182 English practices included in the GPRD which had ‘research standard’ data continuously from 1st April 2005 to 31st March 2008, and which had given consent to link patient data to small area measures of deprivation. The sample was stratified by age, gender and practice. We dropped 8 practices with entirely missing deprivation data. To use the most up-to-date resource use data and the largest possible observation period for diagnoses, we included the 86,100 individuals from the original sample who were alive and registered at one of the remaining 174 practices on 1st April 2007. For the regression analysis we dropped 154 individuals with missing deprivation data.

#### 2.1.3 Costing

We applied national unit costs to the numbers of consultations, prescription drugs, and tests initiated within primary care for each patient to calculate the total cost to the NHS of primary care resources used during the NHS financial year 1st April 2007 to 31st March 2008. All costs were valued in £ sterling at 2007/08 prices. Details of the costing procedures are in the Data Appendix.

#### 2.1.4 Measures of morbidity and multimorbidity

We constructed eight alternative morbidity measures for each patient (Table 1). In addition to measures based on the Quality and Outcomes Framework we chose measures based on the Charlson Index and the John Hopkins ACG system because they are widely used internationally and straightforward to operationalise with routine data.

*QOF diseases*. We used the 17 chronic conditions included in the clinical domain of the 2006/7 version of the Quality and Outcomes Framework which is a pay for performance scheme covering all practices in the UK.^3^ This set of morbidity markers is simple and has high face validity as the main business of general practices is dealing with chronic conditions, although it omits some chronic conditions such as skin disease and liver disease.^4^ The 17 QOF morbidities were included in the regression models as 17 dummy variables. We also use a count of the number of QOF morbidity categories as a multimorbidity measure.

*Charlson diseases*. The Charlson Index is a weighted sum of 17 disease dummy variables selected for their association with mortality (Charlson et al., 1987). About half of the 17 conditions are similar to those in the set of QOF chronic conditions, though the precise definitions vary. As with the QOF diseases, we estimated models with dummy variables for the 17 Charlson diseases and separate models with the Charlson Index as a multimorbidity measure.^5^

The John Hopkins ACG Case-Mix System is also diagnosis-based and was developed using administrative claims data in the United States (Starfield et al., 1991; Weiner et al., 1991). We used the John Hopkins software^6^ to construct four morbidity measures:

*Expanded Diagnosis Clusters* (EDCs) are groupings of clinically similar diagnostic codes. An individual was assigned to an EDC if they had any diagnosis relating to that EDC. We designated 114 of the 264 EDCs as representing a chronic condition (Salisbury et al., 2011) and measured morbidity as a vector of 114 dummy variables. We also counted the number of chronic EDCs in which an individual was included as a measure of multimorbidity.

*Adjusted Clinical Groups* (ACGs) are 68 mutually exclusive categories defined by diagnoses, duration, severity, diagnostic certainty, aetiology, age and gender.^7^ At least 35 ACGs are for multimorbid patients.

*Resource Utilization Bands* (RUBs). The ACG software groups ACGs with similar expected cost into 6 Resource Utilization Bands where higher bands are expected to have higher costs and patients in them are more likely to be multimorbid.

Table 1 summarises the three morbidity measures (vectors of QOF, Charlson and EDC morbidity markers) and the five measures of multimorbidity (counts of the QOF, Charlson, and EDCs markers, plus ACG and RUB categories) used in our analysis. The QOF, Charlson, and EDC morbidity categories were constructed using all historic diagnoses on patients’ general practice records up to 31st March 2007. The ACG and RUB measures use diagnoses over the one-year period 1st April 2006 to 31st March 2007 (John Hopkins Bloomberg School of Public Health, 2008).

#### 2.1.5 Covariates

For each gender, we categorised age at 1st April 2007 into ten-year age bands, with 90+ years as the upper category.

The Index of Multiple Deprivation (IMD) 2007 is a widely used summary measure of deprivation in seven dimensions (income, employment, health and disability, education, housing, environment, crime) for small English areas. It is derived from 38 socioeconomic variables by a complex procedure involving factor analysis, ranking, exponentiation of ranks, and standardisation (Department for Communities and Local Government, 2008). It is calculated at Lower Super Output Area (LSOA)^8^ level. In order to protect patient confidentiality we were provided only with the IMD 2007 decile of the LSOA in which a patient lives.

Because the deprivation measure is ordered and categorical we enter it in the regression models as a set of dummy variables for deciles 2–10 (most deprived). We test for horizontal inequity by examining whether greater deprivation is associated with greater or smaller cost. Because of the ordered and categorical nature of the deprivation variable we do not compute the concentration index which is the standard summary measure of horizontal inequity.

Patient confidentiality also meant that we were provided with anonymised practice identifiers so that we could attach a practice dummy to each patient but had no information on practice characteristics such as the GP to patient ratio.

### 2.2 Modelling

We estimated separate regression models of individual cost using the eight morbidity and multimorbidity measures. The three numerical multimorbidity measures (QOF count, Charlson Index score, EDC count) and the ordered RUB multimorbidity measure were included as dummy variable categories to estimate the most flexible relationships between multimorbidity and cost. In our dataset, the maximum QOF count was 10, the maximum Charlson score was 13, and the maximum EDC count was 28. We used 6 categories for the QOF count (1,2,…,6 or more), 7 for the Charlson (1,2,…,7 or more) and 18 for the EDC count (1,2,…,18 or more) as there were few patients with larger numerical scores. We used dummy variables for the 68 mutually exclusive ACG categories. For the models with non-mutually exclusive QOF, Charlson, and EDC morbidity categories, we used dummy variables for each of the categories.

All the explanatory variables are measured at the start of the cost year 2007/8 with morbidity and multimorbidity variables based on patient morbidity records up to 31st March 2007.

We report results from GLMs in which a link function of the conditional expected 2007/8 cost of the *i*’th patient is linear in the explanatory variables:

(1) $$g(Ec_{iadmp})=\beta_{0}+\sum_{a^{\prime }}\beta_{a^{\prime }}D_{ia^{\prime }}+\sum_{d^{\prime }}\beta_{d^{\prime }}D_{id^{\prime }}+\sum_{m^{\prime }}\beta_{m^{\prime }}D_{im^{\prime }}+\sum_{p^{\prime }}\beta_{p^{\prime }}D_{ip^{\prime }}$$

The *D*_*ia*_ are 15 age/gender group dummies, the *D*_*id*_ are 9 deprivation decile dummies, the *D*_*im*_ are morbidity or multimorbidity category dummies, and the *D*_*ip*_ are 173 practice dummies.

We compare a log link (*g*(*Ec*) = ln *Ec*) with a Poisson (variance equal to the mean) distribution and linear link (*g*(*Ec*) = *Ec*) with a normal distribution. The log form allows for the right skewness of the patient cost data and use of the GLM specification means that we do not have to correct for retransformation bias (Manning, 1998) or adjust the dependent variable because a proportion of patients have zero cost. With a linear link function and a normal error distribution GLM is equivalent to OLS. The log link GLM specification ln *Ec* = *x*′*β* or, equivalently *Ec* = exp(*x*′*β*), is also referred to as an exponential model.

The GLM estimators are consistent and asymptotically normally distributed as long as (1) is valid, but the distributional assumptions need not be correct. For inference, robust standard errors were calculated allowing for a general form of heteroskedasticity and clustering of errors within practices. Models were estimated using STATA 12.1.

We summarise model performance with four goodness of fit measures. The Bayesian Information Criterion (BIC) (Schwarz, 1978) penalises models with more explanatory variables. The deviance-based $R_{D}^{2}$ can be interpreted as the fraction of empirical uncertainty in total patient cost which has been explained by the model (Cameron and Windmeijer, 1997). It is equal to the usual *R*^2^ in an OLS model. Like the BIC its value depends on the assumed error distribution and so cannot be used to compare performance of the log link Poisson GLMs with the OLS models.

$R_{COR}^{2}$ is the squared correlation coefficient between the estimated cost from a model and actual cost. For OLS regression models it is equal to the usual *R*^2^ and hence to $R_{D}^{2}$. We also compute the mean absolute error (MAE), which is the average absolute difference in £’s between observed and estimated cost. $R_{COR}^{2}$ and MAE do not depend on the assumed error distribution and so can be used to compare models with the same set of explanatories but different error distributions.

## 3 Results

### 3.1 Summary statistics

The average total cost per patient was £330 of which 57% arose from prescribed drugs, 35% from consultations, and the remaining 8% from tests and investigations.^9^ Table 2 shows that at most ages women have higher costs but costs increase more rapidly with age for men and are higher for men aged 70–79 and 80–89. The age-gender pattern is similar to those in other UK datasets for the same period (Hippisley-Cox and Vinogradova, 2009; NHS Information Centre, 2008b). Like other healthcare cost data, our primary care cost data are also right skewed (skewness 7.43, with the mean cost being 2.5 times the median), although the proportion of patients with no cost in 2007/8 is smaller (12.3%) than in typical distributions of hospital costs.

### 3.2 Overall model performance

#### 3.2.1 Morbidity measures and model fit

Table 3 shows that the inclusion of any measure of morbidity or multimorbidity boosts the performance of the regression models considerably. For example, with age and gender groups, deprivation deciles, and practice effects the $R_{D}^{2}$ for the log link Poisson GLM specification is 0.23. Adding the set of Charlson indicators, the worst performing of the eight morbidity and multimorbidity measures, to the model increases the $R_{D}^{2}$ to 0.34. Similar increases in performance are seen with the OLS model.

For any given estimation method, the rankings of the eight morbidity and multimorbidity measures by the BIC, $R_{D}^{2}$, $R_{COR}^{2}$ and MAE criteria are very similar. In the log link Poisson GLM specification, the EDC count has the best performance on all goodness of fit statistics closely followed by the set of 114 EDC indicators. These two EDC based measures are noticeably better than the QOF count, 68 ACG indicators, 17 QOF disease categories, and the 6 RUBs, which in turn are markedly better than the Charlson Index score and 17 Charlson disease categories.

Under OLS estimation, the EDC indicators have the best performance followed by the QOF indicators and then the EDC count. The three sets of morbidity category dummies (EDC, QOF, Charlson) performed better than the corresponding count multimorbidity measures.

It is notable that using a simple count of EDC diagnoses as a measure of multimorbidity does better than the more complex set of ACG categories which were designed to describe multimorbid patients. The ACG categories also have a worse overall performance than the count of QOF diseases.

#### 3.2.2 Comparison of estimation methods

For the models without any morbidity or multimorbidity measures the log link Poisson models and OLS models have very similar performance in terms of MAE and $R_{COR}^{2}$ for any given set of covariates. With any of the morbidity or multimorbidity measures, both the log Poisson GLM and OLS estimation methods have good explanatory power, comparable with similar types of studies of primary care costs. However, the relative performance of the two estimation methods is dependent on the morbidity measure used. The log link Poisson GLM has lower MAE and higher $R_{COR}^{2}$ than OLS for three of the eight models with morbidity or multimorbidity measures, OLS does better for three, and there is essentially no difference for two models.

Our results contrast somewhat with previous comparisons of OLS and GLM models for hospital costs (Gravelle et al., 2011; Van de Ven and Ellis, 2000). This may be because our sample is small relative to these studies,^10^ though our data have a smaller proportion of zero cost patients than is usual in hospital cost studies. However, the difference in explanatory power is not large and OLS estimation with the 114 EDC categories had the lowest MAE and highest $R_{COR}^{2}$ over all sets of explanatory variables and estimation methods.

### 3.3 Morbidity measures: distributions and cost ratios

Tables 4–6 show the percentage of patients in different morbidity categories for each of the 8 morbidity and multimorbidity measures and also the effect on cost of being in those morbidity categories.

The distribution of the EDC count has a larger range than the QOF chronic disease count and Charlson Index score because of the greater number of relatively minor diseases that the EDC count includes. According to the QOF chronic disease count 20% of patients were multimorbid (had a count of two or more) whereas 61% were multimorbid according to the EDC count. Women had slightly higher scores than men on the three count multimorbidity measures (QOF count, Charlson Index score, EDC count). There were significant positive Spearman rank correlations amongst these three count measures (for the top censored counts used in the models) – QOF and Charlson: 0.63; QOF and EDC: 0.72; Charlson and EDC: 0.59.

There are differences across the QOF, Charlson and EDC morbidity categorisations in the proportions of patients with some of the diseases. For example, 14.3% of patients have asthma in the EDC scheme but only 6.5% in the QOF scheme. The QOF payments for asthma patients relate mainly to the monitoring of patients and therefore patients require a recent inhaler prescription to be classified as asthmatic, whereas EDC requires only a diagnosis of asthma. The QOF distinguishes between asthma and chronic obstructive pulmonary disease and so only records 2.1% of patients as having chronic obstructive pulmonary disease, whereas the Charlson scheme records 16.6% as having chronic pulmonary disease because its definition includes asthmatics.

Tables 4–6 give cost ratios for the morbidity categories from the log link GLM model which estimates ln *Ec* = *x*′*β* or *Ec* = exp(*x*′*β*). Since all explanatory variables are binary, the cost ratio for a variable *x*_*k*_ is the ratio of expected cost when *x*_*k*_ = 1 to the expected cost when *x*_*k*_ = 0 and is exp(*β*_*k*_ * 1 + ∑_*j*≠*k*_*β*_*j*_*x*_*j*_)/exp(*β*_*k*_ * 0 + ∑_*j*≠*k*_*β*_*j*_*x*_*j*_) = exp *β*_*k*_.

For multimorbidity measures (QOF count, Charlson count, and EDC count, ACGs, RUBs) which place patients in mutually exclusive categories, the cost ratio is relative to a zero count or to non-users. For example, in Table 5, a patient with an EDC count of 4 has an expected cost which is 6.13 times as large as a patient with a zero EDC count. For the three sets of non-mutually exclusive morbidity dummies (QOF diseases, Charlson diseases, EDCs) the cost ratio is the cost of the disease relative to not having that disease, rather than to not having any disease. For example, in Table 4, a patient diagnosed as having cancer under the QOF scheme has an expected cost 1.74 times as large as the average patient without cancer (including those with other QOF diagnoses or no QOF diagnosis).

Amongst the QOF morbidity categories epilepsy is the chronic disease with the biggest relative effect (2.32 compared with no epilepsy), though only 0.9% of the sample have the condition. The most common QOF condition is hypertension (18.2% of the sample) with a cost ratio of 1.42. All the QOF disease cost ratios are significantly greater than 1 but their range is limited (1.09–2.32). The 17 Charlson diseases also have a similarly limited range of cost ratios (0.98–2.43).^11^

Of the 114 EDCs cost ratios, 82 are significant. All but 8 are less than 2.0 and the largest (Transplant status) has a cost ratio of 3.6 (but only 0.05% of patients are in this category). Table 5 reports some examplar categories.

The baseline ACG category is non-users and all other categories have a cost ratio in excess of 1. The results for selected ACGs in Table 6 suggest that in general patients with more morbidities have higher costs. Cost ratios increase with RUB levels in the table, which also suggests that multimorbid patients are more costly as patients in higher RUBs are more likely to be multimorbid.

The cost ratios for the three count measures increase with the counts (except for EDC counts 14 and 17, which are slightly smaller, respectively, than EDC counts 13 and 16) implying, like the ACG and RUB results, that patients with more diseases have higher costs. We re-estimated the QOF count and EDC count log Poisson models with the counts and their squares rather than with categories for the counts. We found that the estimated proportionate effect of an additional disease on the cost ratio declines with the number of diseases. Plots of predicted costs against the EDC count, QOF count, Charlson score and RUB level (see Brilleman et al., 2011) suggests a roughly linear, rather than exponential, effect of increasing multimorbidity on the level of costs.

### 3.4 Practice effects

Including fixed practice effects in the models contributes little to model performance. Adding practice effects to models with only age and gender or only age, gender and deprivation increases model $R_{D}^{2}$ by at most 0.01. Dropping all the practice fixed effects from the full EDC count model reduces the $R_{D}^{2}$ by 0.02.

The practice dummy variables pick up the effects of characteristics of practices such as the GP to patient ratio, idiosyncratic practice treatment styles, and differences in the practice means of both observed and unobserved patient characteristics. The small impact of including practice dummies suggests that there is little cross practice variation in the means of observed variables, and that there is little cross practice variation in the unobserved practice level factors or that they have little effect.

### 3.5 Age and gender

When morbidity measures are included in the cost model the qualitative pattern of the unconditional age and gender means in Table 2 is maintained but the effects of age and gender on cost are greatly reduced. For example, the conditional effect of being male aged 80–89 is 2.38 times as large as being male aged 20–29 according to the log Poisson model with EDC morbidity categories, whereas the ratio of the unconditional mean costs is 9.13. Older patients are more costly mainly because they are sicker than younger patients.

### 3.6 Deprivation and horizontal inequity

Table 3 shows that adding the measure of patient socioeconomic status (small area deprivation decile) to models with age and gender (Model 2 vs Model 1) or models with age, gender and practice effects (Model 4 vs Model 3) leads to only modest improvements in model fit. Adding the deprivation measure to models with morbidity has similarly small effects.

In all the models, with or without morbidity measures, patients in higher deprivation deciles have greater cost (Table 7). The unconditional cost ratio between the 10th and 1st deprivation deciles is 1.50 and all the conditional cost ratios between the 10th and 1st deprivation decile from models which include a morbidity measure are also statistically significantly greater than 1.00. Our results suggest that there is horizontal pro-poor inequity in primary care even after allowing for rich clinical morbidity measures.

Previous studies have found that the positive relationship between low socioeconomic status and utilisation of primary care is weakened by allowing for self reported patient morbidity and by other methods of reducing unobservable patient heterogeneity. With EDC indicators the estimated cost ratio is reduced from 1.50 to 1.15 and 6 of the 9 ratios of cost relative to cost in the lowest decile are not significantly different from 1.00 at the 5% level. Table 7 shows that including other detailed clinical morbidity measures also reduces the cost ratio between the 10th and 1st deciles. In general, the better fitting is the model, the smaller is the cost ratio between most and least deprived deciles.

### 3.7 Sensitivity analyses

There were some patients with extremely high costs: the highest four costs ranged from £15,128 to £27,810 compared to the median cost of £134. The use of the log link in the GLM models reduced this discrepancy considerably (the log of the highest cost was 10.233 compared to the log of the median of 4.895). Dropping patients above the 99th cost centile (£2471 or 7.812 in logs) led to slight improvements of all models but did not alter their relative performance.

We also estimated GLM models with a log link and gamma error distribution (error variance proportional to the square of the mean). In all cases, the log link gamma models had higher MAE and smaller $R_{COR}^{2}$ than the log link Poisson models. The $R_{COR}^{2}$ was much smaller for the models with EDC indicators (0.03 vs 0.29) and QOF indicators (0.11 vs 0.25).

We experimented with a more elaborate method of measuring multimorbidity using the QOF disease categories, rather than using a simple count. We added 136 dummy variables for each pair wise combination of the 17 QOF categories to the OLS model with the 17 QOF categories. This led to only a small improvement in the model *R*^2^ from 0.314 to 0.324 compared to the simpler model with 17 QOF disease categories.^12^

## 4 Capitation payments and morbidity measures

Most primary care capitation systems compute capitation payments for patients using only mean costs by age and gender. To examine the implications of the availability of detailed morbidity data for capitation payments Table 8 compares capitation payments based only on age and gender (Model 1)^13^ with payments based on regression models which also include patient morbidity measures.^14^ We compute a patient's capitation as their expected cost given their age, gender, deprivation and morbidity. To ensure that a patient's capitation is not affected by which practice they belong to we replace the estimated effect of the patients’ practice with the average of the practice effects. We normalise capitation payments so that they sum to total cost across the whole sample.

Capitation payments for individual patients differ substantially across the six models. On the whole, capitations from models with morbidity measures are more closely correlated with those from other models including morbidity (range 0.617–0.849) than with those based on age and gender only (range 0.490–0.677). There are also generally smaller average absolute differences between capitations based on models including morbidity (range £81 to £154) than between capitations from these models and capitation based only on age and gender (range £134 to £176). These average absolute patient capitation differences across models are large relative to the mean patient cost of £330.

The underlying model used to compute the capitation makes much less of a difference at practice level. To illustrate this we show in the final column the mean absolute differences between total “practice” capitation as a proportion of total cost. Because our 10% random sample of patients in 174 practices has just under 500 patients from each actual practice we group sample patients randomly in 17 artificial “practices” of just over 5000 patients to provide a more realistic illustration of practice level differences. The differences between total payments computed using models with different morbidity measures are small: the largest differences are just over 1%.

One potential problem with capitation regimes is that practices have a financial incentive to select patients if capitation differs from the cost which the practice expects the patient to impose on the practice. The absolute difference between capitation and actual cost for an individual patient is likely to overstate the incentive for selection since some of the future cost of a patient is pure noise and not predictable even with full knowledge of the patient's current medical condition. To illustrate the magnitude of potential incentives for patient selection, we examine the difference between the average capitation payment^15^ for particular types of patient and their average cost.

Table 9 shows the difference between average capitation from different cost models and mean actual cost for various patient types defined by age, gender, diagnoses, and, in some cases, also by deprivation.^16^ Patients with a diagnosis have only that diagnosis. Thus, because there are more EDC diagnoses than QOF diagnoses, we see that there are more women aged 20–29 in the highest deprivation decile who have no QOF disease than have no EDC disease. Conversely, because there are more non-QOF diseases than non-EDC diseases, women aged 20–29 with no QOF disease also have higher average cost than women aged 20–29 with no EDC disease. Capitation based only on age/gender mean costs exceeds the mean cost of patient types with no diagnoses but is much less than mean cost for types with high cost. For some patient types, such as men aged 40–49 with EDC asthma, mean capitation exceeds mean costs for all capitation calculations. For others, mean capitation is less than mean cost by a considerable margin, for example for men aged 50–59 with QOF obesity and diabetes. It is not however necessarily the case that there are bigger differences between mean capitation and mean cost for patient types with high costs. The highest cost type shown (men aged 70–79 with QOF hypertension, CHD and CKD) with a mean cost of £983 has smaller selection incentives for all models than men aged 50–59 with QOF obesity and diabetes who have a substantially smaller mean cost of £644.

Table 9 also illustrates a second potential problem with capitation based on morbidity: it creates incentives to “up-code” i.e. report additional diseases for a patient in order to boost capitation payments.^17^ For example, adding a diagnosis of QOF hypertension for a female patient aged 50–59 with no QOF disease will boost capitation based on QOF disease categories by, on average, £110.^18^ Under QOF count based capitation, payment will increase by £228. Similarly adding a diagnosis of EDC hypertension to a female patient aged 50–59 with no EDC disease will increase capitation by £31 when capitation is based on EDC indicators and £74 when it is based on the EDC count. However, it is not always the case that the gains from up-coding are smaller with the finer EDC disease categorisation. Adding a diagnosis of QOF hypertension for a man aged 40–49 with QOF asthma boosts capitation by £31 under QOF count based capitation, but adding a diagnosis of EDC hypertension for a man aged 40–49 with EDC asthma increases capitation by £66 under EDC count based capitation.

Capitation based only on age and gender creates bigger incentives for patient selection than capitation based on cost models which also include individual morbidity measures. By contrast miscoding is less of a problem under age and gender based capitation since it is easier for funders to audit the age and gender of patients rather than their morbidity.

## 5 Conclusion

### 5.1 Discussion

Adding measures of individual patient morbidity produces a considerable boost in the explanatory power of cost models. Using finer categories of morbidity improves the prediction of patient costs. Thus measures using the 114 EDC categories were better than those using the 17 category QOF scheme, whether the categories were used to measure morbidity as a set of dummy variables or used to construct counts of disease categories to measure multimorbidity. The QOF based measures of morbidity and multimorbidity performed considerably better than the Charlson based measures which had the same number of morbidity categories. The poor performance of the widely used Charlson Index score and of the Charlson disease dummy variables may be because the Charlson scheme was originally intended to predict mortality rather than the cost of general practice activities. The two QOF based measures had about the same predictive power as the 68 mutually exclusive ACG categories derived using purpose built case-mix software. This may be because the 17 QOF categories were selected for a primary care pay for performance scheme targeted at care for chronic patients who are the main business of general practices. The ACG categories included non-chronic diagnoses which were grouped in part by their anticipated effect on all patient costs, including hospital costs.

We found that the morbidity measure had an effect on the relative performance of the OLS and GLM log link Poisson estimators although the differences in performance for a given morbidity measure were not large. The choice of morbidity measure has a bigger impact on model performance than the choice of estimator.

Although we were limited to a fairly crude ordered categorical and summary measure of socio-economic status, our results are in line with the previous literature: more deprived patients had greater cost but the association became weaker when morbidity measures were included in the cost model. The better fitting the cost model, the smaller was the association between deprivation and cost.

A major reason for interest in predicting primary care cost is to inform the calculation of capitation payments. We found that capitation payments for individuals vary considerably with the morbidity system used in the cost models, though the choice of morbidity system had much less effect on total payments to practices.

There were considerable potential capitation increases from up-coding, i.e. from overstating the number of diagnoses for patients when capitation was based in part on morbidity. Conversely, incentives for patient selection were reduced markedly when morbidity information was used to calculate capitation payments. However, even when capitation was based on models including morbidity measures the difference between average capitation and average cost for certain types of patient was still sizeable compared to average patient costs.

The models were estimated on data from a sample of English general practices. However, similar data are increasingly available in other countries and our conclusions, that morbidity measures based on detailed clinical records greatly improve the ability to predict primary care costs but that choice of measure affects both the performance of the cost model and the individual capitation payments based on it, are likely to apply in other health care systems.

The ACG and Charlson morbidity categories were originally derived and calibrated on US data and the QOF categories were developed as part of a UK pay for performance scheme. The institutional differences across countries mean that the choice of morbidity measure for computing capitations requires investigation using country specific data. It would also be useful to examine alternative methods of producing summary morbidity measures from the very detailed raw clinical information on individual patients, including, for example, factor analysis (Fang et al., 2008). These investigations would also be improved by richer socio-economic data than we were able to use and by panel data to allow for unobserved patient factors. Given the importance of prescription costs it may also be useful to examine how much separate modelling of prescribing and other costs would improve predictive power.

### 5.2 Simpler is better?

The best performing multimorbidity measures were simple counts of the number of chronic conditions patients suffered from or simple sets of disease dummies. It is notable that using an 18 category count of EDC diagnoses as a measure of multimorbidity does better than the more complex set of 68 ACG categories which were designed to describe multimorbid patients. The ACG categories also have a worse overall performance than the seven category count of QOF diseases. It is possible that, when morbidity descriptive systems are designed to predict cost in a specific institutional setting, more elaborate schemes will do better than simple ones. But simplicity has other virtues. Simpler morbidity and multimorbidity schemes are easier for patients and GPs to understand. Setting capitation fees based on morbidity requires that patient morbidity be measured every budgetary period for every patient and more complex schemes have higher measurement and computation costs. Thus there may be a trade-off between simplicity and predictive power when alternative morbidity measures are considered.

## Figures and Tables

**Table A1:** Unit costs (£, 2007/08) per primary care encounters.

Staff type	Surgery consultation	Home visit	Clinic	Telephone consultation	Out of hours
GP: partner	24.47	81.37	35.98	14.85	36.97
GP: registrar/associate	15.92	52.92	23.40	9.66	24.05
GP: sole practitioner	27.55	91.61	40.51	16.72	41.63
Practice nurse	9.00	–	9.00	5.46	–
Counsellor	64.00	–	–	–	–
Other health care professional	15.00	–	15.00	9.10	–

**Table 1:** Morbidity and multimorbidity measures.

Measure	Number diseases/categories	Range of measure	Details
QOF disease dummy variables	17 Not mutually exclusive	0–1 dummies	17 chronic diseases in the clinical domain of the UK Quality and Outcomes Framework (QOF) pay for performance scheme: asthma, atrial fibrillation, cancer, coronary heart disease (CHD), chronic kidney disease (CKD), chronic obstructive pulmonary disease (COPD), dementia, depression, diabetes, epilepsy, heart failure, hypertension, learning difficulties, mental health, obesity, stroke, and hyperthyroidism.
QOF disease count	17	0–17	Count of the QOF diseases.
Charlson disease dummy variables	17 Not mutually exclusive	0–1 dummies	17 diseases predictive of mortality: cerebrovascular disease (1), chronic pulmonary disease (1), congestive heart disease (1), dementia (1), diabetes (1), mild liver disease (1), myocardial infarction (1), peptic ulcer disease (1), peripheral vascular disease (1), rheumatological disease (1), cancer (2), diabetes with complications (2), hemiplegia and paraplegia (2), renal disease (2), moderate or severe liver disease (3), AIDS (6), and metastatic tumour (6). Not mutually exclusive. (Numbers in parentheses are weights in Charlson Index score – see below.)
Charlson Index score	17	0–33	Weighted count of Charlson diseases. Weights reflect strength of relationship with patient mortality.
Expanded Diagnosis Clusters (EDCs) dummy variables	114 Not mutually exclusive	0–1 dummies	Chronic clinically related groupings of diagnoses.
Count of EDCs	114	0–114	Count of EDCs.
Adjusted Clinical Groups (ACGs)	68 mutually exclusive categories	0–1 dummies	Classification into an ACG based on age, gender, combination of morbidities, and expected cost. The age range of our sample meant we used only 68 out of 82 possible ACG categories.
Resource Utilization Bands (RUBs)	6 mutually exclusive categories	0–1 dummies	ACGs grouped into 6 mutually exclusive Resource Utilization Bands on the basis of expected costs; 0: No or only invalid diagnoses; 1: Healthy Users; 2: Low; 3: Moderate; 4: High; 5: Very High.

**Table 2:** Patient primary care costs (£) 2007/8.

	Age	Number	Mean cost	SD	Median	% with zero cost
Male	20–29	6021	90	367	24	29.4
	30–39	7204	113	300	26	29.9
	40–49	8902	168	524	40	25.9
	50–59	7486	282	530	92	19.7
	60–69	6481	458	622	264	8.0
	70–79	4112	681	722	484	2.5
	80–89	1878	822	783	608	1.9
	90+	253	567	510	428	6.3

Female	20–29	5551	176	239	106	7.0
	30–39	6930	210	407	110	8.0
	40–49	8447	240	429	111	7.0
	50–59	7525	334	515	168	5.0
	60–69	6563	483	636	293	2.6
	70–79	4954	639	662	463	1.6
	80–89	3057	709	772	555	1.3
	90+	736	673	579	528	2.2
All patients		86,100	330	563	134	12.3

*Notes*. Costs are the sum of the costs of prescriptions, tests, face to face or telephone consultations plus the costs of administration for repeat prescriptions and other administration not requiring face to face or phone contact.

**Table 3:** Goodness of fit of alternative specifications of model for total patient cost.

Model specification	Log, Poisson				Linear, Gaussian (OLS)		
	BIC	$R_{D}^{2}$	$R_{COR}^{2}$	MAE	BIC	$R_{COR}^{2}=R_{D}^{2}=R^{2}$	MAE
Age, gender (Model 1)	38446223	0.21	0.13	285	1320939	0.13	285
Age, gender, and deprivation (Model 2)	38041309	0.22	0.13	283	1320555	0.13	284
Age, gender, and practice (Model 3)	37725187	0.22	0.14	282	1322027	0.14	283
Age, gender, deprivation, and practice (Model 4)	37522639	0.23	0.14	281	1321883	0.14	282
(Model 4) + QOF disease indicators	29339460	0.40	0.25	244	1302791	0.31	234
(Model 4) + QOF chronic disease count	28523441	0.42	0.29	239	1305547	0.29	240
(Model 4) + Charlson indicators	32546370	0.33	0.22	259	1310235	0.25	255
(Model 4) + Charlson Index score	32274547	0.34	0.23	258	1311982	0.23	260
(Model 4) + EDC indicators	26255449	0.46	0.29	231	1295660	0.37	222
(Model 4) + EDC count	26196861	0.46	0.32	229	1302546	0.31	236
(Model 4) + ACG	28694630	0.41	0.27	242	1308944	0.27	248
(Model 4) + RUB	30367472	0.38	0.24	250	1312522	0.23	259

*Notes*. BIC: Bayesian Information Criterion. Smaller BIC indicates better fit and is comparable for different models with same error distribution. $R_{D}^{2}$: deviance based *R*^2^, which is comparable for different models with same error distribution. $R_{COR}^{2}$: squared correlation coefficient from OLS regression of estimated cost on actual cost. For OLS models $R_{COR}^{2}$, the deviance based $R_{D}^{2}$, and the model *R*^2^ are equal. MAE: mean absolute error. Estimation sample: 85,946 patients aged 20+ in 174 practices.

**Table 4:** Patients and cost ratios for QOF disease categories, QOF disease count, Charlson disease categories, and Charlson Index score.

QOF disease categories				QOF disease count				Charlson disease categories				Charlson Index score			
Disease	% pats.	Cost ratio	95% CI	Count	% pats.	Cost ratio	95% CI	Disease	% pats.	Cost ratio	95% CI	Score	% pats.	Cost ratio	95% CI
None	55.06	1.00	–	0	55.06	1.00	–	None	68.84	1.00	–	0	68.84	1.00	–
Asthma	6.52	1.80	(1.74,1.86)	1	24.81	2.41	(2.34,2.48)	AIDS	0.01	0.98	(0.51,1.88)	1	19.27	2.05	(2.00,2.10)
Atrial fibrillation	2.11	1.11	(1.06,1.17)	2	11.14	3.80	(3.67,3.93)	Cancer	3.83	1.54	(1.48,1.60)	2	6.62	2.59	(2.50,2.68)
Cancer	1.64	1.74	(1.65,1.84)	3	5.20	4.86	(4.68,5.04)	Cerebrovascular	2.31	1.32	(1.25,1.39)	3	2.98	3.16	(3.02,3.31)
CHD	5.63	1.45	(1.41,1.50)	4	2.39	5.81	(5.55,6.07)	Chronic pulmonary	16.63	1.64	(1.61,1.68)	4	1.30	3.60	(3.42,3.80)
CKD	3.84	1.15	(1.10,1.20)	5	0.92	7.27	(6.85,7.73)	Congestive heart	1.21	1.24	(1.17,1.32)	5	0.56	3.86	(3.58,4.16)
COPD	2.09	1.64	(1.56,1.72)	6+	0.49	7.56	(7.04,8.11)	Dementia	0.41	1.29	(1.16,1.44)	6	0.23	4.13	(3.70,4.62)
Dementia	0.48	1.27	(1.15,1.41)					Diabetes	4.28	1.98	(1.92,2.05)	7+	0.19	4.54	(3.98,5.18)
Depression	14.87	1.53	(1.49,1.57)					Diabetes with comp.	0.91	2.43	(2.27,2.61)				
Diabetes	5.04	1.73	(1.67,1.79)					Hemiplegia/paraplegia	0.18	1.86	(1.46,2.36)				
Epilepsy	0.93	2.32	(2.13,2.53)					Metastatic tumour	0.12	1.36	(1.07,1.71)				
Heart failure	1.15	1.09	(1.02,1.17)					Mild liver disease	0.17	1.39	(1.16,1.67)				
Hypertension	18.16	1.42	(1.38,1.46)					Mod/sev liver disease	0.04	2.11	(1.32,3.38)				
Learning	0.37	1.70	(1.41,2.05)					Myocardial infarction	1.70	1.42	(1.35,1.50)				
Mental health	0.91	2.06	(1.88,2.26)					Peptic ulcer	2.15	1.31	(1.25,1.38)				
Obesity	9.92	1.30	(1.26,1.34)					Periph vascular dis.	1.43	1.26	(1.20,1.33)				
Stroke	2.42	1.22	(1.16,1.27)					Renal disease	4.68	1.34	(1.28,1.40)				
Hyperthyroidism	3.91	1.21	(1.16,1.26)					Rheumatological dis.	2.05	1.46	(1.39,1.53)				

*Notes*. Estimates from GLM log Poisson model also including age/gender, deprivation and practice effects. Cost ratios for disease categories are the estimated costs for a patient with the relevant disease divided by the estimated cost for a patient without that disease. Cost ratios for counts are the estimated costs for a patient with the relevant count divided by the estimated cost for a patient with no disease (zero count). CHD: coronary heart disease; CKD: chronic kidney disease; COPD: chronic obstructive pulmonary disease; AIDS: acquired immune deficiency syndrome.

**Table 5:** Patients and cost ratios for selected EDC categories and EDC count.

Selected EDC categories				EDC count			
Category	% patients	Cost ratio	95% CI	Count	% patients	Cost ratio	95% CI
None	19.39	1.00	–	0	19.39	1.00	–
Low back pain	25.80	1.10	(1.08,1.13)	1	19.56	2.11	(2.01,2.21)
Dermatitis and eczema	19.57	1.12	(1.09,1.15)	2	16.36	3.34	(3.20,3.48)
Hypertension	18.36	1.38	(1.35,1.42)	3	12.54	4.64	(4.44,4.85)
Anxiety, neuroses	16.50	1.21	(1.18,1.24)	4	9.20	6.13	(5.85,6.42)
Depression	16.21	1.29	(1.26,1.33)	5	6.75	7.73	(7.37,8.11)
Asthma	14.26	1.50	(1.46,1.54)	6	4.90	9.08	(8.65,9.53)
Cervical pain syndromes	13.20	1.10	(1.07,1.12)	7	3.51	11.01	(10.40,11.66)
Arthritis	11.17	1.13	(1.09,1.16)	8	2.41	12.16	(11.49,12.86)
Irritable bowel syndrome	6.78	1.19	(1.14,1.23)	9	1.74	13.74	(12.92,14.61)
Gastroesophageal reflux	6.70	1.27	(1.23,1.31)	10	1.25	15.02	(14.13,15.97)
Acute myocardial infarction	5.84	1.26	(1.22,1.30)	11	0.82	16.96	(15.81,18.20)
Malignant neoplasm of the skin	2.53	1.07	(1.02,1.12)	12	0.57	17.18	(15.89,18.58)
Malignant neoplasms, breast	1.09	1.56	(1.46,1.68)	13	0.36	19.52	(17.87,21.32)
Emphysema, chron bronchitis, COPD	2.38	1.30	(1.24,1.36)	14	0.27	19.05	(17.17,21.13)
				15	0.15	21.04	(18.65,23.73)
				16	0.09	24.69	(21.43,28.46)
				17	0.07	24.00	(19.98,28.83)
				18+	0.08	27.60	(24.08,31.63)

*Notes*. Estimates from GLM log Poisson model also including age, gender, deprivation and practice effects. Cost ratios for disease categories are the estimated costs for a patient with the relevant disease divided by the estimated cost for a patient without that disease. Cost ratios for counts are the estimated costs for a patient with the relevant count divided by the estimated cost for a patient with no disease (zero count).

**Table 6:** Patients and cost ratios for selected ACG categories and for Resource Use Bands.

Selected ACG categories				RUB			
Category	% patients	Cost ratio	95% CI	Band	% patients	Cost ratio	95% CI
Non-users	9.44	1.00	–	Non-user	9.44	1.00	–
No diagnosis or only unclassified Diagnosis	20.05	2.58	(2.40,2.77)	Healthy user	36.36	3.28	(3.06,3.51)
Preventive/administrative	6.47	4.04	(3.72,4.39)	Low morbid	25.03	5.60	(5.22,6.01)
Acute minor, age 6+	9.63	4.25	(3.95,4.57)	Moderate	27.26	9.54	(8.90,10.23)
Chronic medical: stable	2.49	7.06	(6.49,7.68)	High	1.70	13.44	(12.36,14.62)
2–3 Other ADG combinations, age 35+	9.98	8.93	(8.31,9.60)	Very high	0.20	16.02	(13.81,18.58)
4–5 Other ADG combinations, age 45+, no major ADGs	1.83	10.90	(10.05,11.82)				
4–5 Other ADG combinations, age 45+, 1 major ADG	2.28	12.50	(11.56,13.51)				
6–9 Other ADG combinations, age 35+, 0–1 major ADG	1.52	16.32	(15.03,17.71)				
10+ other ADG combinations, age 18+, 2 major ADGs	0.05	21.75	(16.90,27.99)				
6–9 other ADG combinations, male, age 18–34, 1 major ADG	0.01	46.78	(20.99,104.22)				

*Notes*. Estimates from GLM log Poisson model also including age, gender, deprivation and practice effects. Cost ratios for mutually exclusive ACG categories (or RUBs) are the relevant model estimated costs for a patient in the ACG (or RUB) category divided by the estimated cost for a patient with no use. ADGs: Adjusted Disease Groups are combinations of diagnoses used to construct Adjusted Clinical Groups.

**Table 7:** Effect of deprivation on cost with different morbidity measures.

Model	Model $R_{COR}^{2}$	Proportionate difference in cost between 10th and 1st deprivation decile	95% CI
Age/gender, deprivation, practice (Model 4)	0.14	1.50	[1.40,1.60]
Model 4 + QOF indicators	0.25	1.19	[1.12,1.27]
Model 4 + QOF count	0.29	1.20	[1.13,1.28]
Model 4 + Charlson indicators	0.22	1.33	[1.24,1.41]
Model 4 + Charlson Index	0.23	1.34	[1.26,1.43]
Model 4 + EDC indicators	0.29	1.15	[1.09,1.23]
Model 4 + EDC count	0.32	1.22	[1.15,1.29]
Model 4 + ACG categories	0.27	1.33	[1.25,1.42]
Model 4 + RUB	0.24	1.38	[1.30,1.47]

*Notes*. All models are estimated as GLM log link, Poisson. OLS results are similar.

**Table 8:** Capitation payments with different morbidity measures and estimation methods.

Model	vs Model	Individual patient capitation		Practice capitation
		Correlation coefficient	Mean patient absolute difference between capitation payments (£s)	Mean total absolute practice difference as a proportion of practice total cost
Age and gender only	QOF disease indicators	0.571	134.3	0.0044
	QOF count	0.660	162.2	0.0085
	EDC indicators	0.490	158.3	0.0053
	EDC count	0.621	175.8	0.0106
	ACG	0.677	153.0	0.0078
QOF disease indicators	QOF count	0.849	80.5	0.0068
	EDC indicators	0.746	104.1	0.0068
	EDC count	0.697	148.9	0.0112
	ACG	0.636	150.0	0.0112
QOF count	EDC indicators	0.694	124.9	0.0087
	EDC count	0.794	136.9	0.0091
	ACG	0.732	148.4	0.0056
EDC indicators	EDC count	0.803	101.4	0.0070
	ACG	0.617	153.9	0.0062
EDC count	ACG	0.756	148.6	0.0100

*Notes*. Capitation payments calculated from age/gender only are the age/gender means. All other capitation payments are calculated from log Poisson models containing age/gender, deprivation decile, practice effect and morbidity, but with average practice effects replacing the estimated effect of the patient's practice. Practice differences are computed by randomly assigning patients to 17 “practices” of around 5056 patients. Capitation payments calculated from OLS models give similar results.

**Table 9:** Difference between capitation and average cost for selected patient groups.

Patient type	*N* patients	% of sample	Mean cost	Average capitation from model minus mean cost					
				Age/gender mean	QOF indicators	QOF count	EDC indicators	EDC count	ACG
Female, 20–29, deprivation decile 10, no QOF disease	546	0.64%	145	31	14	−13	21	14	33
Female, 20–29, deprivation decile 10, no EDC disease	198	0.23%	103	73	60	43	25	−40	39
Female, 50–59, no QOF disease	3787	4.41%	175	159	−15	46	42	51	84
Female, 50–59, QOF hypertension	439	0.51%	350	−16	−19	38	−10	26	11
Female, 50–59, no EDC disease	1003	1.17%	91	243	79	133	−25	78	100
Female, 50–59, EDC hypertension	113	0.13%	155	179	96	70	45	−15	88
Male, 40–49, QOF asthma	270	0.31%	270	−101	−63	−6	−62	−29	−53
Male, 40–49, QOF asthma and hypertension	21	0.02%	548	−379	−231	−129	−258	−215	−250
Male, 40–49, EDC asthma	188	0.22%	102	67	46	37	31	11	46
Male, 40–49, EDC asthma and hypertension	10	0.01%	155	14	13	22	6	24	24
Male, 50–59, QOF obesity & diabetes	49	0.06%	644	−363	−213	−77	−213	−171	−231
Male, 70–79, QOF hypertension & CHD	110	0.13%	755	−74	−83	66	−61	−3	3
Male, 70–79, QOF hypertension, CHD & CKD	30	0.03%	983	−302	−27	73	16	−29	−96
Male, 20–29, EDC dermatitis/eczema & anxiety/neuroses	28	0.03%	106	−16	−11	−6	−7	15	2
Female, 40–49, EDC IBS	63	0.07%	122	118	75	58	43	0	66
Female, 40–49, EDC IBS & depression	28	0.03%	201	39	20	24	−4	−8	26
Female, 20–29, deprivation decile 10, ACG category: No Diagnosis or Only Unclassified Diagnosis	106	0.12%	131	45	35	46	19	45	−36
Male, 70–79, ACG category: Chronic Medical: Stable	161	0.19%	620	61	150	158	−17	92	33

*Notes*. Capitation payments calculated from age/gender only are the age/gender means. All other capitation payments are calculated from log Poisson models containing age/gender, deprivation decile, practice effect and morbidity, but with average practice effects replacing the estimated effect of the patient's practice. Capitation payments calculated from OLS models give similar results. IBS: irritable bowel syndrome.

## Appendix A Data Appendix

We calculated the total cost of primary care resources used by each patient during the NHS financial year 1st April 2007 to 31st March 2008 by applying national average unit costs to the numbers of consultations, prescription drugs, and tests initiated within primary care. All costs were valued in £ sterling at 2007/08 prices.

Consultations included all face-to-face (including surgery consultation, home visit, clinic, out of hours) and telephone consultations. The unit cost of each consultation was based on a combination of consultation type and primary staff role (type of general practitioner, practice nurse or other health care professional leading the consultation). The unit costs for consultations are shown in Table A1. They include costs of administrative activities such as the recording of results or sending mail to a patient when this was performed by a receptionist, administrator, or secretary. Unit costs were taken from Curtis, 2008 and from a report on national average GP earnings and expenses based on GP tax returns (Technical Steering Committee, 2010). The unit costs in Curtis, 2008 are derived by estimating a national average annual cost for each type of staff, dividing by the national average number of hours worked per year, and combining this cost per hour with an estimated average encounter time for each type of staff. The costs include salaries, training costs, and premises.

Although payments to a practice vary with performance under the QOF and with the prevalence of QOF diseases in the practice, the unit costs used to cost primary care activity in a particular practice are based on national average payments to GPs and so are not affected by the prevalence of QOF diseases in that practice.

Unit costs for prescription drugs were based on information provided by the GPRD which combined data from several sources, including the National Drug Tariff for generic products, and manufacturers for branded products. Each prescription drug in the patient level data was matched to unit cost using drug name, strength and formulation. Where there was more than one unit cost for a prescription drug we used the median unit cost.

To allow for possible ambiguities in the mapping of recorded drug quantities to costs we also computed a measure of prescription costs using data from the prescription cost analysis of the NHS, 2008a which provides information on the net ingredient cost of all prescriptions dispensed in the community in England. We used the British National Formulary (BNF) code (BRM, 2010) to attach these average costs to each prescription in the GPRD data, ignoring the quantity specified in the prescription. Our results are not sensitive to the method for calculating prescribing costs. Using national average amounts per prescription rather than recorded amounts to calculate total primary care costs reduced model performance somewhat but estimated effects were similar and the model rankings were unchanged. Ambiguities in the recording of the amount of a given drug prescribed will have only small effects on practice capitation based on the coefficients from estimated cost models and will not create incentives for misreporting of morbidity.

Estimated drug costs use data on prescriptions issued by the practice, not on drugs actually dispensed to patients, as NHS data systems for dispensing are primarily used to remunerate pharmacists. Not all prescriptions issued to patients result in the dispensing of medicines. One study estimated that 5.2% of prescriptions written in a general practice were not dispensed (Beardon et al., 1993). Our drug costs are therefore an overestimate. Whether this results in biased estimates of the effects of morbidity and other variables depends on whether the propensity to have prescriptions dispensed is correlated with the explanatory variables.

With advice from a general practitioner member of the research team (SP) we determined which tests were performed within a standard surgery consultation and applied a zero unit cost, save for the cost associated with any consumables such as pregnancy test kits or urine dipsticks. Unit costs for the remaining tests were based on the National Health Service (NHS) Reference Costs (Department of Health, 2009). For laboratory tests the unit cost was based on pathology discipline. Hospital-based tests and investigations requested by the practice were costed using the NHS Reference Costs.

We included cost data for patients who were alive and registered with our sample practices on 1st April 2007 at the start of the financial year 2007/8. We made no adjustment for patients who deregistered from the practice list during the resource use year. Thus if patients moved to another practice during the year, their total primary care costs defined to include those incurred in other practices, would be under recorded in our data. If the aim is to estimate future costs which will be incurred by the practice in which the patient is initially registered this does not present a problem. On average, setting capitation fees based on our results would ensure that practices would be paid for costs actually incurred by patients on their list at the start of the year. A retrospective adjustment could be made at the end of the financial year for costs attributable to newly registered patients. We also make no adjustment for patients who die. Such patients may have higher costs but to the extent that the morbidity measures are correlated with mortality in the coming year the estimated effects of different types of morbidity will include some of the differentially higher costs of patients at higher risk of death.

## Footnotes

1 The electronic record systems in UK general practices use 85,000 Version 2 Read codes to record clinical information. The Read codes can be mapped into ICD-10 which has over 9000 diagnostic categories in its finest classification (excluding chapters XX and XXII). See http://www.who.int/classifications/icd/en/.

2 Previous comparisons of the predictive power of alternative morbidity and multimorbidity schemes have focussed on hospital cost (Huntley et al., 2012; Winkelman and Mehmud, 2007; Perkins et al., 2004).

3 We used the QOF Business Rules Version 16 which outlines the clinical Version 2 Read codes and any additional criteria required to include a patient on the relevant QOF disease register: NHS Primary Care Contracting. QOF Implementation Business Rules v16. http://www.primarycarecontracting.nhs.uk/145.php.

4 Although reported disease prevalence affects the total QOF payment, Gravelle et al. (2010) found no evidence that practices gamed their reporting of the prevalence of QOF diseases, possibly because of the auditing of QOF data by local primary care organisations.

5 We use an adaptation by Khan et al. (2010) which provides the clinical Version 2 Read codes for diagnosing each disease, using a translation from the widely used Deyo adaptation of the Charlson Index for ICD-9 codes (Deyo et al., 1992).

6 We used the John Hopkins ACG System Version 8.2 (Johns Hopkins Bloomberg School of Public Health, 2008) to obtain the EDC classifications.

7 Though the ACG System Version 8.2 identifies up to 82 default ACGs (or up to 93 with optional branching), the age range of our sample meant that some of these categories were not populated.

8 There are 32,482 LSOAs in England with a mean population of 1500.

9 Fuller summary statistics on cost are in Brilleman et al. (2011).

10 In Gravelle et al. (2011) models were estimated on samples of around 5M patients.

11 Patients with AIDS do not increase general practice costs significantly because in the UK their care and the prescribing of AIDS drugs are generally managed in secondary care.

12 See Brilleman et al. (2013) for a discussion of the cost implications of particular pairwise combinations of QOF conditions.

13 In the English NHS capitation fees are based on the results from two studies of the determinants of consultation rates and consultation length, as measures of GP workload. The study of consultation length did not include any morbidity variables and the consultation rate study was based on area level data and used area measures of mortality and of patient reported morbidity (Formula Review Group, 2007). Funding for general practice prescribing is allocated by a formula derived from a practice level model of prescribing costs which included three practice level disease prevalence measures (Department of Health, 2011). Data confidentiality meant that we could not identify practices and so could not compare capitation based on morbidity with actual NHS capitation payments. Given the specificities of the NHS capitation system, a comparison with simple age and gender based capitation has more general lessons.

14 To save space we do not report results with the three worst performing morbidity measures (Charlson indicators, Charlson Index, RUBs).

15 When the definition of a patient type does not depend on deprivation, patients of that type will have capitation which varies with their deprivation.

16 Since practice effects had little explanatory power and do not greatly affect estimated coefficients on morbidity and other explanatory variables, the capitation payments are very similar to predicted cost. Thus the differences between mean costs and capitations also provide an indication of how well the model estimates costs for particular patient types.

17 Most health care systems have a large enough number of practices to ensure that no single practice would be able to influence the estimated model coefficients, and hence the capitation formula, by strategic reporting.

18 Under the QOF indicators model the capitation for a women aged 50–59 with no QOF disease is £221 (mean cost £175, plus the £46 excess of average capitation over mean cost) and for a woman aged 50–59 with QOF hypertension it is £331 (mean cost £350 plus the −£19 excess of average capitation over mean cost).
